# Solar radiation and surface temperature datasets across urban surface materials of Jakarta megacity

**DOI:** 10.1016/j.dib.2026.112982

**Published:** 2026-06-17

**Authors:** Faradiba, Muh Taufik, Yonny Koesmaryono, Perdinan, Impron

**Affiliations:** a Department of Geophysics and Meteorology, Kampus IPB Darmaga, IPB University, Bogor 16680, Indonesia; b Physics Education Study Program, Universitas Kristen Indonesia, Jakarta 13630, Indonesia; c Centre for Environmental Research, Kampus IPB Dramaga, IPB University, Bogor 16680, Indonesia

**Keywords:** Solar radiation, Surface temperature, Urban materials, Surface albedo, field measurement

## Abstract

Surface characteristics play a crucial role in surface temperature dynamics and the urban heat island phenomenon, yet field-based dataset relates to them is still limited, particularly in tropical urban environments. This article describes data of solar radiation (incoming and outgoing) and surface temperature from various urban materials. We collected data at 5-minute intervals for 30–120 min from fifteen urban surface types of Jakarta Megacity, Indonesia to represent their different reflective and thermal responses. An experimental data describing surface albedo in the field is presented as well. The dataset demonstrates the different variations in radiation reflection for each material, which has implications for the potential heat storage. This data can be used for surface thermal response analysis, evaluation of urban heat island mitigation strategies, development of high-albedo materials, and urban microclimate modelling in megacity. Further, the dataset provides an empirical basis for further studies on sustainable urban design and adaptive surface material planning to high radiation conditions.

Specifications Table

**Table:** 

Subject	Earth & Environmental Sciences
Specific subject area	Urban climate and Urban Heat Island (UHI) dynamics
Type of data	Table, csv file
Data collection	Data of solar radiation and surface temperature were collected through in situ measurements for various urban surface materials. We used automatic sensors that were connected to an Arduino-base IoT system for automatic recording. The measurements were at 5-minute intervals for 30 – 120 min for each surface material depending on weather condition.
Data source location	Jakarta, Indonesia (coordinate: 6°11′ S 106^,0^50’E)
Data accessibility	Repository name: Mendeley Data Data identification number: 10.17632/gw9t6d7by2.1 Direct URL to data: https://data.mendeley.com/datasets/gw9t6d7by2/2
Related research article	None

## 1 Value of the Data

• This dataset presents high-resolution field measurements (5-minute intervals) that provide quantitative information on variations in reflectance and thermal response of materials, supporting studies of urban climate and urban heat mitigation.
• This data can be used to evaluate how surface thermal characteristics affect thermal comfort in urban areas. This information is important for designing more heat-adaptive public spaces, pedestrian paths, and residential areas.
• This data can support the evaluation and development of cooling materials and green infrastructure strategies, including reflective surfaces and urban vegetation integration.
• This data is useful for researchers in the development and evaluation of urban environmental models, and helps to understand the dynamics of the interaction of radiation and surface temperature in more depth.

## 2 Background

Urban development increases the use of hard materials such as asphalt and concrete, which alter the characteristics of the land surface and energy balance [1]. These changes primarily affect radiant reflectance and heat storage. Albedo is a key parameter that determines the amount of reflected radiation [2]. Materials with low albedo absorb more energy, thus increasing surface temperatures in urban areas [3].

Most studies have monitored and evaluated surface temperature and albedo based on remote sensing approaches [4,5], yet, the availability of field-based dataset that simultaneously remains limited, particularly in developing tropical regions. Ground-based measurements are essential for validating surface energy balance models, calibrating satellite estimates, and analysing the dynamic thermal response of materials [6].

As a metropolitan area, Jakarta megacity has experienced increasing surface temperatures influenced by the dominance of low-albedo built-up materials [7]. An intense year-round solar radiation exposure, combined with its humid tropical climate, makes this region a representative location for empirically evaluating the reflectance properties and thermal response of various urban surface materials. Therefore, this study aims to generate a high-resolution field dataset that includes incoming radiation, reflected radiation, albedo values, and surface temperature for various types of urban materials.

## 3 Data Description

**Fig. 1:**
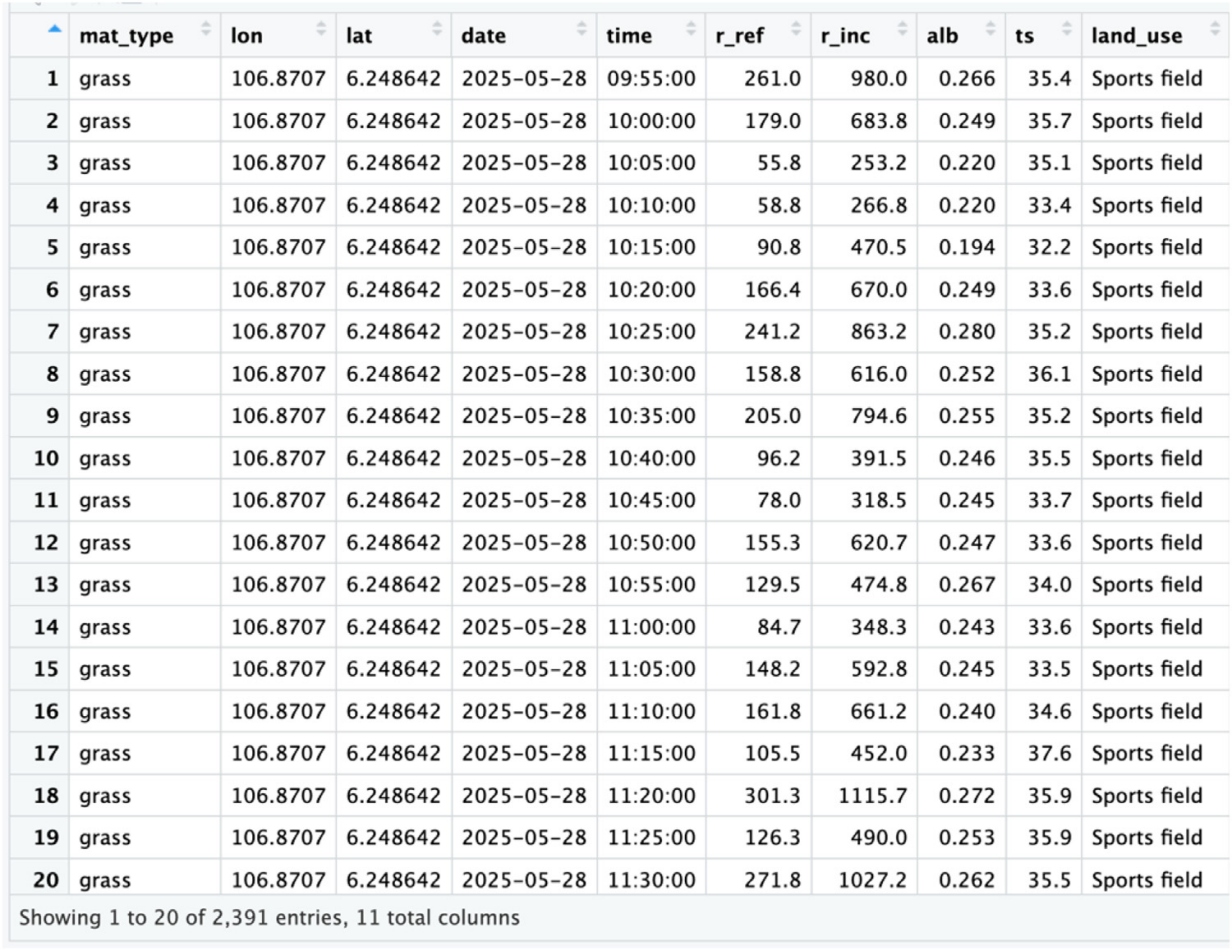
Example view of the raw dataset in .csv format, showing timestamp, geographic coordinates, incoming radiation, reflected radiation, albedo, surface temperature, and land-use attributes for each observation record.

This study produced a field dataset on the radiation reflectance and surface temperature characteristics of various urban materials. The dataset is stored in a single .csv file, which includes information on material type, geographic coordinates (longitude and latitude), measurement date and time (timestamp), incoming radiation (Wm⁻²), reflected radiation (Wm⁻²), albedo value, surface temperature ( °C), and land use information (Table 1). The dataset is organized in a time series format with a date-time index and spatial attributes for each data record (Fig. 1) [8]. Data analysis was performed using the R programming language for statistical analysis and visualization [9].

**Table 1:** Attribute description.

Field	Description
mat_type	Types of urban surface materials measured
lon	The longitude coordinates of the measurement location in decimal degrees (°).
lat	Latitude coordinates of the measurement location in decimal degrees (°).
date	Measurement date (format:YYYY-MM-DD).
time	Measurement time (format: HH:MM:SS).
r_ref	Shortwave radiation reflected by surfaces (W m⁻²).
r_inc	Incoming shortwave radiation received by the surface (W m⁻²).
alb	The ratio between reflected radiation and incoming radiation (-)
ts	Near surface temperature of material at measurement time ( °C)
land_use	Land use categories at the measurement location
weather	Weather conditions during the measurement process

**Fig. 2:**
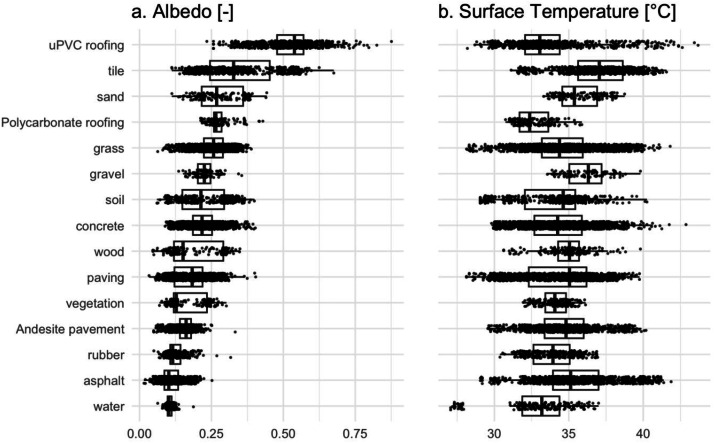
Distribution of albedo (a) and near-surface temperature (b) measured across 15 urban materials in Jakarta. Boxplot represent the median, interquartile range, and data dispersion derived from the raw time-series observations summarized in Table 2.

Table 2 and Fig. 2 present the temporal variation of albedo and surface temperature values for each urban material. The graph illustrates the differences in shortwave reflectance properties between the materials measured over the observation period.

**Table 2:** Summary statistics of albedo and near-surface temperature derived from the raw data observations for fifteen urban surface materials.

Surface type	Albedo mean	Albedo range	Ts mean	Ts range	N observation
water	0.105	0.063 - 0.187	32.8	27.06 - 37.00	47
asphalt	0.111	0.016 - 0.252	35.5	29.06 - 41.88	282
rubber	0.126	0.050 - 0.317	33.8	30.38 - 36.94	80
Andesite pavement	0.157	0.049 - 0.333	34.7	29.56 - 40.19	191
vegetation	0.166	0.069 - 0.302	34.1	31.94 - 36.12	53
paving	0.178	0.033 - 0.404	34.4	28.12 - 39.75	343
wood	0.193	0.046 - 0.349	34.9	30.62 - 39.81	48
concrete	0.218	0.089 - 0.403	34.3	29.81 - 42.88	344
soil	0.223	0.061 - 0.399	33.9	29.05 - 40.25	124
gravel	0.227	0.136 - 0.355	36.2	33.56 - 39.81	21
grass	0.254	0.063 - 0.389	34.6	28.13 - 41.81	429
Polycarbonate roofing	0.276	0.212 - 0.429	32.7	30.75 - 35.88	36
sand	0.277	0.113 - 0.442	35.7	33.31 - 38.75	41
tile	0.344	0.112 - 0.675	37.7	31.12 - 41.56	133
uPVC roofing	0.523	0.234 - 0.876	33.7	28.19 - 43.69	219

## 4 Experimental Design, Materials and Methods

### 4.1 Study area and data collection

**Fig. 3:**
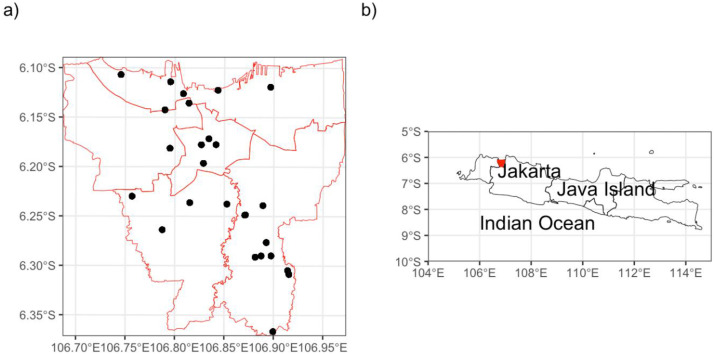
Geographical location of study area in Java Island: (a) spatial distribution of locations for field measurement in Jakarta megacity (b) Study area within Java Island.

Measurements were carried out in the urban area of Jakarta megacity, Indonesia, which has a humid tropical climate with high solar radiation intensity throughout the year. The annual rainfall is about 2000 mm/year [10], which is slightly lower than rainfall in equatorial region [11]. Locations for measurements were selected to represent various types of urban surface materials commonly found in urban environment (Fig. 3), such as grass, asphalt, concrete, paving blocks, and several types of roofs. The geographic coordinates (longitude and latitude) of each measurement point were recorded using a GPS-enabled smartphone application.

Measurements were carried out from May to December 2025, with data collection taking place between 8:00 AM and 5:00 PM local time. Data collection was performed only during sunny or rain-free weather conditions and were therefore adjusted based on meteorological conditions that supported stable solar radiation and optimal representation of surface temperature.

Totally 25 measurement points were purposively selected to represent the variety of surface material types commonly found in urban environments (Table 3, Fig. 3). Measurements were carried out in situ using instruments capable of recording incoming radiation, reflected radiation, and near-surface temperature.

**Table 3:** Distribution of measurement locations.

Location	Measurement point	Materials
West Jakarta	3	Andesite pavement, concrete, asphalt, paving, grass, rubber, vegetation.
East Jakarta	9	Grass, paving, asphalt, uPVC roofing, rubber, soil, grass, concrete, tile, polycarbonate roofing
South Jakarta	4	Andesite pavement, gravel, grass, wood, soil, sand, concrete, paving.
North Jakarta	5	Wood, paving, sand, andesite pavement, water, grass, paving, soil, tile, concrete.
Central Jakarta	4	Paving, tile, grass, concrete, andesite pavement, ruber, water, vegetation, paving, asphalt.

### 4.2 Experimental design

**Fig. 4:**
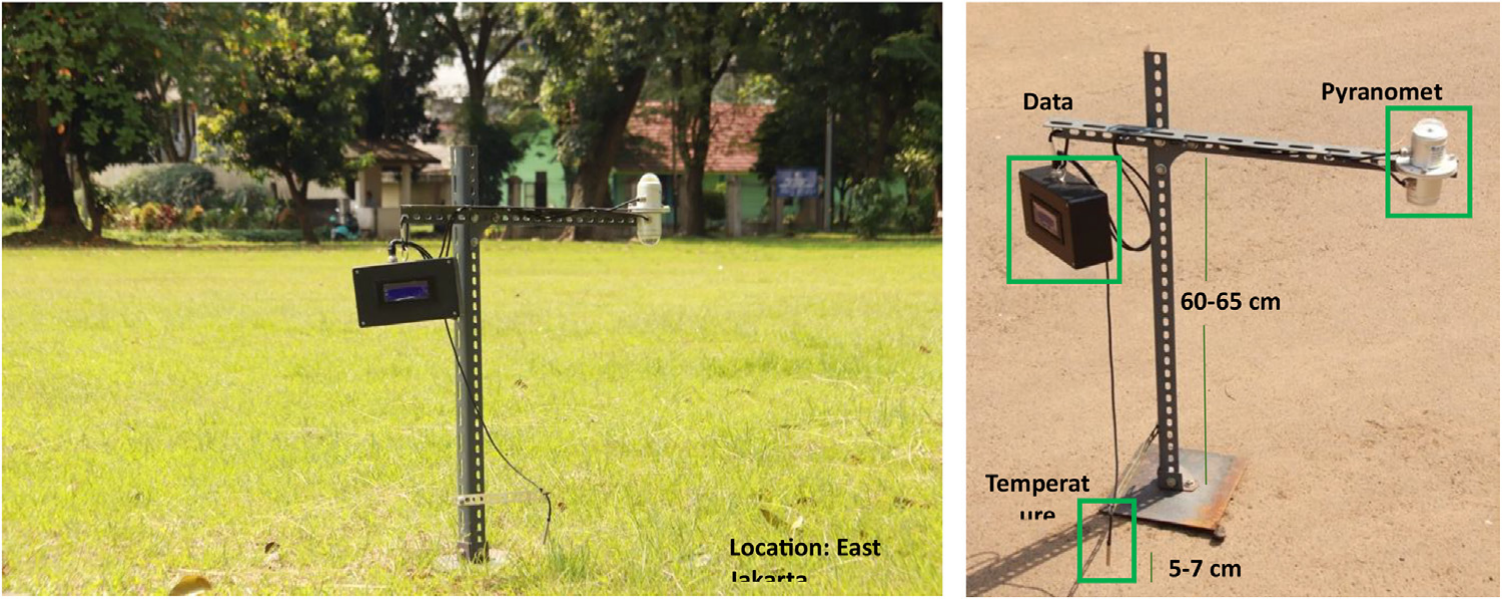
Field setup of the radiation and surface temperature measurement system installed at the study site.

For each surface material, observations were carried out up to 120 min under rain-free conditions. Some field observations were shortened due to unexpected rainfall or rapidly changing atmospheric conditions, but we keep the duration of observation at least 30 min. Observed data were recorded at 5-minute intervals. The pyranometer, which measures incoming and reflected radiation, were positioned steadily and perpendicular to the surface, at a height of approximately 60–65 cm above the material being measured [12]. The surface temperature sensors were positioned approximately 5–7 cm above the material surface to consistently represent the surface layer temperature (Fig. 4). All sensors were mounted securely to ensure consistency and minimize measurement errors. Measurements were conducted under rain-free weather conditions and predominantly clear-sky environments. This approach was adopted to minimize the influence of precipitation, surface wetness, and transient atmospheric variability in radiation and surface temperature measurements.

The albedo value was calculated as the ratio of reflected radiation to incoming radiation as in Eq. (1):

(1) $$\alpha =\frac{R_{reflected}}{R_{incoming}}$$

where: $R_{reflected}$ is the reflected shortwave radiation (Wm⁻²) and $R_{incoming}$ is incoming shortwave radiation (Wm⁻²).

### 4.3 Instrumentation and data acquisition

Shortwave radiation was measured using two Sentec SEM228A RS232 Solar Radiation Sensor pyranometers (Chengdu Sentec Technology Co., China), with a measurement range of 0–1800 Wm^-2^ and accuracy of ±3%. Both sensors have been calibrated and tested under identical exposure conditions to ensure a measurement consistency, before being used in the field. One sensor measured incoming radiation, and the other reflected radiation from the material's surface. Surface temperature was measured using a DS18B20 waterproof temperature sensor, placed directly on the material's surface.

All sensors were connected to an Arduino microcontroller equipped with WiFi connectivity. This system is an Internet of Things (IoT)-based data acquisition system integrated with the Thinger.io cloud platform for real-time data transmission, storage, and monitoring. Data was automatically recorded every minute using a digital data logger and saved in .csv format.

## Limitations

There are several limitations related to field measurements in this study. First, the dataset is limited by short observation period (30–120 min per material), so the results may not fully capture daily or seasonal variations. Second, the types of materials are limited by availability at the study site, so generalization to other regions with different climate characteristics and urban morphology should be done with caution.

## Ethics Statement

This study does not involve human subjects, animal experimentation, or the use of personal or sensitive data. The dataset was obtained from in situ environmental measurements. The authors confirm compliance with the ethical standards and submission guidelines of Data in Brief.

## CRediT Author Statement

**Faradiba:** Methodology and writing – original draft; **Muh. Taufik:** Conceptualization, supervision, validation and writing – review & editing; **Yonny Koesmaryono:** Writing – review & editing; **Perdinan:** Writing – review & editing; **Impron:** Writing – review & editing.

## Data Availability

Mendeley DataField dataset of radiation and surface temperature across urban surface materials (Original data).
